# Identification of OXA-23 carbapenemases: novel variant OXA-239 in *Acinetobacter baumannii* ST758 clinical isolates in Mexico

**DOI:** 10.1002/nmi2.60

**Published:** 2014-09-22

**Authors:** E M Tamayo-Legorreta, U Garza-Ramos, H Barrios-Camacho, A Sanchez-Perez, A Galicia-Paredes, A Meza-Chavez, J Silva-Sanchez

**Affiliations:** 1 Departamento de Diagnóstico Epidemiológico, Centro de Investigaciones sobre Enfermedades Infecciosas, Instituto Nacional de Salud PúblicaCuernavaca, Morelos, Mexico; 2 Hospital de Oncología, IMSS-CMN-Siglo XXIMexico City, Mexico; 3 Hospital de Cardiología, IMSS-CMN-Siglo XXIMexico City, Mexico

**Keywords:** β-lactam antibiotics, carbapenem resistance, clonal dissemination, multidrug resistance, nosocomial infection

## Abstract

A collection of 15 carbapenem-resistance *Acinetobacter baumannii* clinical isolates was analysed on two tertiary hospitals in Mexico. The OXA-51 was identified in all isolates, followed by OXA-239 and OXA-58; OXA-239 is described as a new OXA-23-like allele. These carbapenemases were identified on four clonal groups, distributed between two neighbouring hospitals. *Acinetobacter baumannii* is poorly studied in Mexico; this situation urges the implementation of strategies to prevent its dissemination.

Dear Editor,

In the past two decades, *Acinetobacter baumannii* has become a major pathogen, responsible for nosocomial infections. This pathogen displays high carbapenem resistance due to the production of carbapenemases, including the enzymes that contribute to the resistance to carbapenems, carbapenem-hydrolysing class D β-lactamases. Four groups have been identified: *bla*OXA-51-like and three acquired ones (*bla*OXA-23-like, *bla*OXA-24-like and *bla*OXA-58-like) 1, however OXA-23 enzymes are found worldwide 2. This study describes the characteristics of carbapenem-resistant *A. baumannii* clinical isolates in two tertiary-care hospitals in Mexico City.

A collection of 15 non-duplicate *A. baumannii* clinical isolates (one from each patient) was included. All of them were imipenem-resistant and so were the cause of nosocomial infections. They were collected between August and December 2010 at two hospitals belonging to the Centro Medico Nacional Siglo-XII (CMN-XXI): Oncology (ten isolates) and Cardiology (five isolates). The main isolation site corresponded to tracheal aspirates (46.6%; 7/15). The bacterial identification was carried out using API 20NE, and antimicrobial susceptibility was determined with the Phoenix system (Becton Dickinson Company, Sparks, MD), using the combined ID and AST NMIC/ID 104 panel for Gram-negative bacilli. The MIC to imipenem, meropenem, tigecycline and colistin were determined using the broth microdilution method following the CLSI recommendations 3. All isolates were resistant to ampicillin, ceftazidime, ciprofloxacin, imipenem and meropenem, but they were susceptible to tigecycline (0.25 μg/mL) and displayed a decreased susceptibility to colistin (2–4 μg/mL) (Table 1). Clonal relatedness was determined by pulsed field gel electrophoresis and analysed according to the criteria proposed by Tenover *et al*. 1995 4, using the gelcompar II software (Applied Maths, Sint-Martens-Latem, Belgium). Four clonal groups (A–D) were identified: Clone A included five clinical isolates from the Cardiology hospital; the other minor clonal groups were obtained from the Oncology hospital (Table1). The multilocus sequence typing (MLST) 5, was carried out in 8407 (clone A) and 8509 (clone B) isolates and the analysis performed on the *Acinetobacter* MLST website, (http://pubmlst.org) showed that the sequence type (ST) was 758 (Table1), corresponding to a new ST.

**Table 1:** Characteristics of carbapenem-resistant *Acinetobacter baumannii* clinical isolates

Strain	Hospital	PFGE	MLST (ST)	OXA-type	IMP	MEM	TIG	CL
8402	1	A	ND	51	32	16	<0.125	4
8404	1	A	ND	58, 51	32	16	<0.125	4
8400	1	A	ND	51	8	16	0.25	4
8407	1	A	758	239, 58, 51	32	32	0.5	4
8406	1	A	ND	51	32	32	0.5	4
8511	2	B	ND	239, 58, 51	32	8	1	4
8513	2	B	ND	51	32	8	1	2
8509	2	B	758	239, 51	32	8	1	4
8510	2	B	ND	239, 51	32	8	1	4
8508	2	B	ND	51	32	8	1	2
8506	2	C	ND	51	32	8	1	2
8516	2	C	ND	51	32	16	0.5	2
8517	2	C	ND	51	32	16	1	2
8514	2	D	ND	51	32	8	1	2
8515	2	D	ND	51	32	8	1	4

Abbreviations: CL, colistin; IMP, imipenem; MEM, meropenem; MLST, The MultiLocus Sequence Typing; ND, not determined; PFGE, pulsed field gel electrophoresis; ST, sequence type; TIG, tigecycline.

Hospitals: 1, Cardiology; 2, Oncology from Centro Medico Nacional Siglo-XII (CMN-XXI) in Mexico City.

The phenotypic detection of metallo-β-lactamase production was achieved by means of a disc approximation screening test 3 and through a PCR assay for VIM, IMP, GIM, SPM and NDM-1 genes, using generic primers as previously reported 6,7; all the results were negative. To detect the class D carbapenemases (OXA-51, OXA-58, OXA-23 and OXA-24), a PCR assay was performed using specific primers 7. All PCR-positive products were purified using a High Pure PCR Product Purification Kit (Roche Applied Science, Indianapolis, IN, USA); they were sequenced using the chain termination method with a Big-Dye Terminator kit (Applied Biosystems, Foster City, CA, USA) and analysed on an ABIPRISMA 3100 (Applied Biosystems). The nucleotide sequences were compared with the GenBank database by means of BLASTx searches. The OXA-51 gene was identified in all isolates, and in three and four isolates, respectively; OXA-58 and/or OXA-23-like genes were detected. In the OXA-23-like gene, three mutations were identified: S109L, D222N and P225S, corresponding to a new allele, OXA-239 (GenBank JQ837239) (http://www.lahey.org/Studies/) (Table1). The genetic context of the OXA-type genes was analysed by PCR mapping, using IS*AbaI* and OXA-23 primers 8. The results showed that IS*AbaI* was not associated to the OXA-51 gene, whereas the IS*AbaI* sequence is flanking the OXA-239 gene, suggesting that its expression could be driven by the promoter present in this insertion sequence, as previously reported 9.

A new OXA-235 allele was described recently in one *A. baumannii* clinical isolate in Mexico 10, this study described another new OXA-23-like allele, OXA-239 gene in *A. baumannii* ST758 clinical isolates with different clonal origin from two tertiary-care hospitals from the same hospital complex in Mexico City. *Acinetobacter baumannii* has been poorly studied in Mexico; however, these studies show OXA enzyme diversification, highlighting the emergence of the new OXA-239 and for this and other reasons it is necessary to implement strategies to identify these kinds of isolates and prevent their dissemination.
